# Supplementary material for: The interactome of intact mitochondria by cross-linking mass spectrometry provides evidence for coexisting respiratory supercomplexes
Source: Mol Cell Proteomics. 2017 Dec 8;17(2):216–32. doi: 10.1074/mcp.RA117.000470 (PMC5795388; doi:10.1074/mcp.RA117.000470)
Supplement: Supplemental Data [file supp_RA117.000470_133922_0_supp_23978_fzffwf.zip › spectra_annotation/mito_spectra_annotation/165-1-9-1-10-1.pdf]

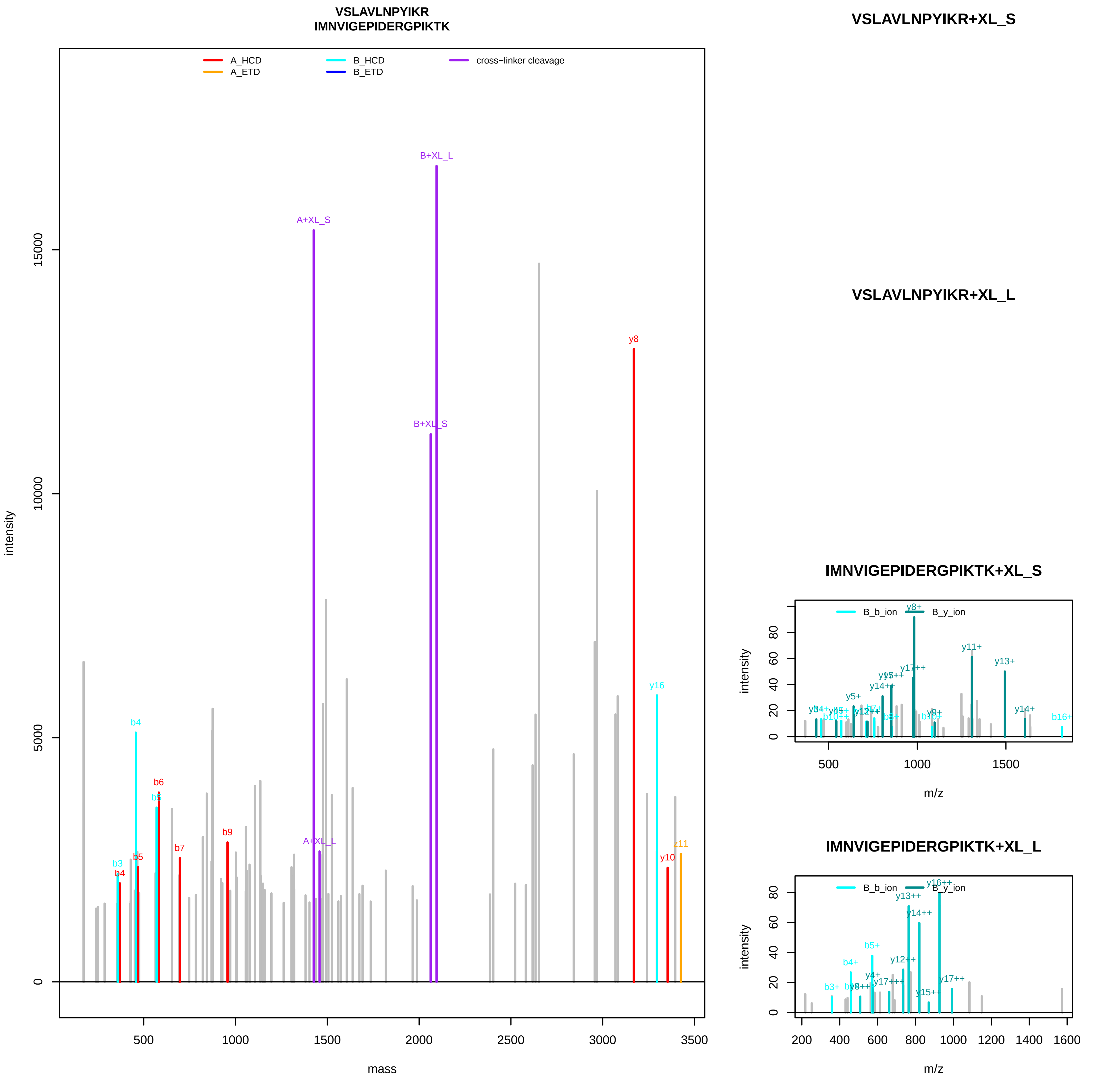
VSLAVLNPYIKR
IMNVIGEPIDERGPIKTK
A_HCD
A_ETD
B_HCD
B_ETD
cross−linker cleavage
B+XL_L
A+XL_S
B+XL_S
A+XL_L
y8
y16
y10
z11
b3
b4
b5
b6
b7
b9
intensity
mass
0
5000
10000
15000
500
1000
1500
2000
2500
3000
3500
VSLAVLNPYIKR+XL_S
VSLAVLNPYIKR+XL_L
IMNVIGEPIDERGPIKTK+XL_S
B_b_ion
B_y_ion
y8+
y11+
y13+
y17++
y14++
y5+
y9+
y14+
b16+
intensity
m/z
0
20
40
60
80
500
1000
1500
IMNVIGEPIDERGPIKTK+XL_L
y16++
y13++
y14++
b5+
b4+
y12++
y4+
b3+
y8++
y17+++
y15++
y17++
200
400
600
800
1000
1200
1400
1600
